# Treatment of War Wounds of the Elbow

**Published:** 1919-01

**Authors:** 


***Treatment of War Wounds of the Elbow.*** By M. A. MARTIN. *La Presse Médicale*, October 3, 1918.

Ten cases of war wounds of the elbow, combined with fractures of that joint, have been treated by M. Martin. The most conservative modes of operation were employed as often as possible. In all cases wide arthrotomy was practised; the bony lesions were curetted carefully, levelled with the gouge forceps, and swabbed with ether. Two cases of fissures of the humeral diaphysis were totally sutured. The quadriceps was always restored as thoroughly as possible by means of a frank, but limited, excision and the subsequent suturing of the muscle and its aponeurotic expansion.

Quite remarkable results have been obtained; all these wounded having recovered the normal or very nearly normal use of the injured joint.

Such examples prove again that typical resection should be strictly limited to such fractures of the joints as cannot be given conservative treatment.

Primary osteosynthesis in cases of fractures of the joints permits, in some instances, the total or sub-total anatomic restoration of the joint.

